# Current Management and Unmet Needs in Alopecia Areata: Viewpoint From a Malaysian Perspective

**DOI:** 10.2196/89583

**Published:** 2026-09-21

**Authors:** Azura Mohd Affandi, Adawiyah Jamil, Noor Zalmy Azizan, Felix Boon-Bin Yap, Kin Fon Leong, Agnes Yoke Hui Heng, Siti Nuraihan, Wooi Chiang Tan, Latha Selvarajah, Teeba Raja, Evelyn Wen Yee Yap, Peter Wee Beng Ch'ng, Tze Yuen Teoh, Mei Ying Tey

**Affiliations:** 1 Department of Dermatology, Ministry of Health, Hospital Kuala Lumpur, Kuala Lumpur, Malaysia; 2 Faculty of Medicine, University Kebangsaan Malaysia, Kuala Lumpur, Malaysia; 3 Central Dermatology Specialist Clinic, Kuala Lumpur, Malaysia; 4 Sunway Medical Centre, Selangor, Malaysia; 5 Dermatology Unit, Hospital Tunku Azizah, Kuala Lumpur, Malaysia; 6 Agnes Heng Dermatology, Ipoh, Malaysia; 7 Pediatric Dermatology Unit, Pediatric Department, Ministry of Health, Hospital Selayang, Selangor, Malaysia; 8 Department of Dermatology, Ministry of Health, Hospital Pulau Pinang, Pulau Pinang, Malaysia; 9 Department of Dermatology, Ministry of Health, Hospital Sultan Ismail, Johor Bahru, Malaysia; 10 Department of Dermatology, Ministry of Health, Hospital Selayang, Selangor, Malaysia; 11 Department of Dermatology, Mahkota Medical Centre, Melaka, Malaysia; 12 Department of Dermatology, Gleneagles Hospital, Kuala Lumpur, Malaysia; 13 Medical Affairs, Pfizer, Level 10 & 11, Wisma Averis, Tower 2, Avenue 5, Bangsar South, No 8, Jalan Kerinchi, Kuala Lumpur, Malaysia

**Keywords:** alopecia, alopecia areata, Malaysia, reimbursement, Janus kinase inhibitors, JAK inhibitors

## Abstract

Alopecia areata (AA) is a chronic, immune-mediated disease characterized by nonscarring hair loss and a substantial clinical and psychosocial burden, yet it is still widely perceived as a purely cosmetic concern. In this viewpoint, we draw on the available Malaysian studies, selected international evidence, and our collective clinical experience to propose that AA warrants greater clinical, research, and policy attention in Malaysia. Malaysia-specific evidence remains limited and is derived largely from retrospective, single-center studies; nonetheless, the available data point to variable disease presentation, inconsistent use of standardized severity assessment, reliance on conventional therapies, substantial loss to follow-up, and a meaningful psychosocial impact among affected patients. Current local management appears to rely mainly on conventional therapies, while access to advanced therapies such as Janus kinase inhibitors remains constrained by affordability and reimbursement challenges. We therefore call for 5 priorities: strengthening Malaysia-specific epidemiological and real-world evidence, including through a national AA registry; adopting standardized disease-assessment tools in clinical studies and routine practice; integrating psychosocial support into AA care pathways; developing locally relevant clinical guidance; and improving public, clinician, and policymaker awareness that AA is a medical condition rather than a cosmetic concern. Together, these steps would support earlier diagnosis, more equitable access to effective treatment, and holistic, patient-centered care for adults and children living with AA in Malaysia.

## Introduction

Alopecia areata (AA) is a chronic autoimmune condition marked by nonscarring hair loss, primarily affecting the scalp, face, and body [1]. The disorder arises when the immune system mistakenly targets active hair follicles, resulting in sharply defined patches of hair loss without permanently damaging the follicular structure. Although many patients may experience spontaneous hair regrowth, AA often follows a relapsing course requiring continuous medical management [2]. AA affects approximately 1 in every 1000 individuals and carries a lifetime risk of around 2% [2]. The condition may occur across all age groups; however, it is somewhat more frequent in children, with AA rates of 1.92% compared with 1.47% in adults [3].

The condition is frequently associated with comorbidities, including vitiligo, lupus, psoriasis, atopic dermatitis, and thyroid disorders [2]. Additionally, other comorbidities such as metabolic syndrome, iron deficiency anemia, vitamin D deficiency, psychiatric diseases, audiologic, and ophthalmic abnormalities are more prevalent in patients with AA, who also have a higher risk of developing other autoimmune conditions [4].

The average age of onset of AA is approximately 30 years, and patients with severe AA tend to have a longer disease duration [5]. Male , nail involvement, and disease duration longer than 1 year are identified as significant risk factors for severe AA [5]. Although AA is not life-threatening, its psychological impact is significant, and it can lead to anxiety disorder, depression, and stress, often necessitating consultation with mental health professionals [6,7]. Anxiety is significantly increased in patients with AA, who are reported to have 2.5 times higher odds of experiencing anxiety compared with control groups [3]. Children and adolescents with AA are equally affected [8], with much greater susceptibility to bullying [9], and recent research indicates that 51% of pediatric patients meet the criteria for an anxiety disorder and 62.9% show at least one psychiatric condition [10].

Malaysia, an upper-middle-income nation with a population of approximately 34 million, is strategically situated in Southeast Asia as a regional hub for information and communication technology and medical tourism [11]. In Malaysia, patients with AA may initially present to primary care settings, including government health clinics (Klinik Kesihatan), private general practitioners, and aesthetic physicians. While some patients with mild, limited hair loss may be managed initially with topical corticosteroids, mild disease may also warrant dermatology referral for diagnostic confirmation, assessment of disease activity, and specialist management. Referral to dermatology is particularly important in cases of diagnostic uncertainty, extensive or rapidly progressive disease, inadequate response to initial treatment, or when systemic treatment is being considered. The public health care system follows a structured referral pathway from primary care to dermatology departments in major hospitals, whereas patients willing to pay out of pocket may access private dermatologists directly. Despite this structure, there is a notable gap in epidemiological data, comprehensive assessments of disease burden, and locally tailored clinical guidelines for AA in Malaysia. Given the significant impact of AA on quality of life and overall health, this Viewpoint aims to consolidate current knowledge on AA epidemiology, clinical burden, and management practices in Malaysia and to set out the key unmet needs within the Malaysian context. Through this Viewpoint, we aim to inform health care professionals, researchers, and policymakers, and to guide future research and health care initiatives that enhance care for patients with AA.

## Opportunity to Strengthen Malaysia-Specific Evidence Base for AA

**Figure 1.**
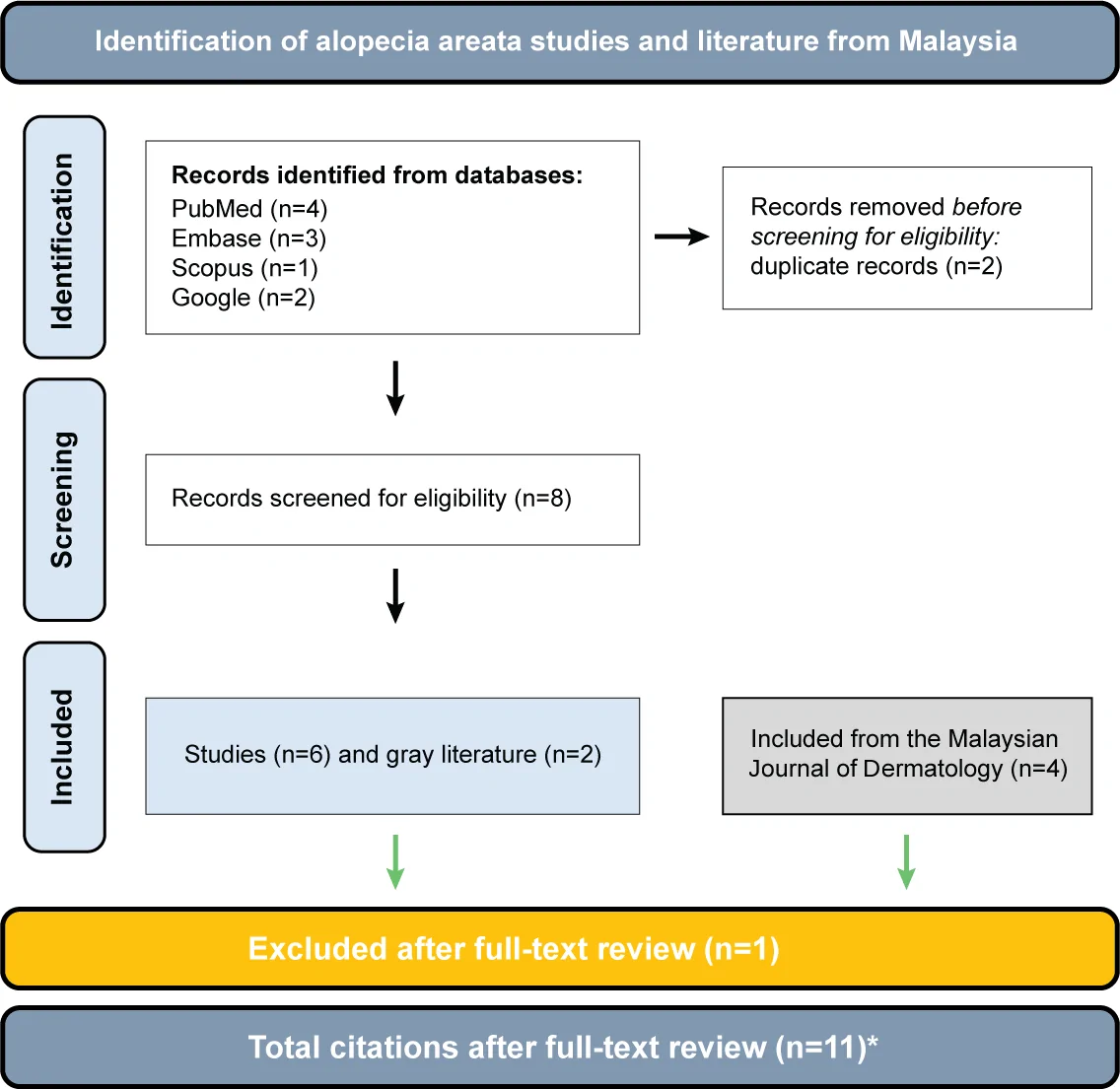
Literature search for the identification of alopecia areata studies from Malaysia. The search used the keywords “alopecia areata” and “Malaysia,” and databases were searched through March 2025. *One citation did not include patients from Malaysia and was excluded after full-text review [23].

AA has been briefly studied in Malaysia across multiple patient cohorts, providing insight into the disease’s demographic and ethnic distribution [12-22] (Figure 1; Multimedia Appendix 1). A study conducted at a tertiary dermatology center in Kuala Lumpur analyzed 200 patients with AA [15]. The findings from this cohort revealed a median age of onset of 25 years, with a male-to-female ratio of 1.38:1. The median duration from the onset of symptoms to presentation was 3 months. The frequency of AA in a dermatology outpatient clinic at Hospital Kuala Lumpur in Kuala Lumpur, Malaysia, was 1.32% in 58,252 patients seen over 7 years from 2008 to 2014 [19]. Another study from the Universiti Malaya Medical Centre reviewed the medical records of 154 patients with nonscarring alopecia (28.6% with AA) from 2000 to 2009 [16,22]. This study reported a mean age at presentation of 24.5 (range 4‐62) years, with 30.1% of patients aged <18 years and 20.5% aged <16 years [16,22]. The male-to-female ratio in this study was nearly equal, at 1:1.05 [16].

The ethnic distribution across the studies was variable but relatively balanced, with most studies showing a higher frequency in Malays, followed by Indians. Heah et al [19] found that Malays represented 69.2% of AA cases, followed by Indians 28% and Chinese 10%, while Yin and Chew [22] reported the highest proportion of patients as Malays (34.1%), followed by Indians (31.8%), and Chinese (22.7%). However, Tang et al [15] reported that Indians had the highest frequency of AA, accounting for 35.5% of cases. Meanwhile, the study by Lee and Lee [16] found that Malays and Indians each represented 32.7% of cases. However, ethnic variations reported in these retrospective Malaysian AA cohorts may also reflect the catchment populations of individual hospitals rather than actual national distributions. Moreover, these frequencies were not reported against the background of Malaysia’s overall ethnic composition, and as such, the degree to which certain ethnic groups are overrepresented is not discernible.

AA exhibits a range of distinct clinical patterns that can vary in severity and progression [3]. Recognizing these different presentations is essential for accurate diagnosis, prognosis, and management, as they may reflect underlying differences in disease activity or response to treatment. Tang et al [15] have best characterized clinical presentations at Kuala Lumpur Hospital in Malaysia: 98.5% (197/200) of patients presented with patchy scalp involvement—often extending to eyebrows, eyelashes, or facial hair—while ophiasis was seen in 3 cases. More extensive disease was uncommon, with alopecia totalis in 0.5% and universalis in 1.0% of patients [15].

On the basis of the available Malaysian studies, existing local evidence is derived mostly from single-center cohorts rather than nationwide data sources. Current evidence warrants further characterization of national prevalence, incidence, severity distribution, treatment patterns, long-term outcomes, and patient-reported burden. Future priorities may include establishing national AA registries to capture robust epidemiological data and the clinical spectrum of disease, to better define the national disease burden, and to support clinical care, treatment decisions, and guideline development.

## Standardize Disease-Assessment Tools in Clinical Studies and Routine Clinical Use

Various tools have been developed to quantify AA severity, and accurate assessment is essential for monitoring progression and guiding treatment [24]. The Severity of Alopecia Tool (SALT), first proposed in 1999, is widely used in clinical trials to quantify hair loss [24,25]. Other objective assessments, such as body surface area (BSA), are also critical for determining eligibility for systemic treatments [26].

While some Malaysian studies have incorporated disease-assessment measures, these have varied across studies, limiting standardization and comparability of disease severity and treatment outcomes. Albela et al [14] categorized the extent of hair loss and regrowth based on assessment criteria by Olsen et al [27], using the SALT score, and defined severe AA as involvement of more than 50% of the total scalp area (S3-S5). Ang and Tang [20] adopted the clinical response measurement by McDonald Hull and Norris grading system. Grades 1 and 2 were considered no response, while grades 3 and 4 were considered a good response. In Kaur [18], disease activity was measured using the hair pull test. The absence of a consistent severity scoring system may hinder accurate disease monitoring, treatment response evaluation, and the development of localized clinical guidelines.

Given the potentially high burden of AA, accurate diagnosis and assessment of disease severity are important to guide effective intervention [28]. Several formal measures of disease severity are used in AA clinical trials, including clinician-reported outcomes such as Severity of Alopecia Tool (SALT), Eyebrow Assessment Scale (EBA), Eyelash Assessment Scale (ELA), Alopecia Areata Investigator Global Assessment (AA-IGA), and patient-reported outcomes such as Alopecia Areata Patient Priority Outcome (AAPPO) and Patient’s Global Impression of Change (PGI-C) [27,29-31].

**Figure 2.**
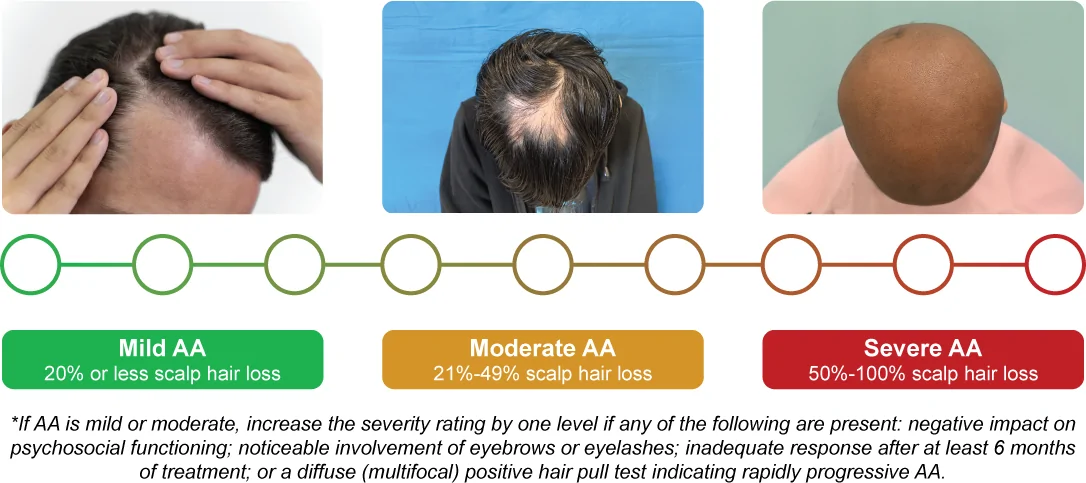
The Alopecia Areata Severity Scale for disease assessment in routine clinical practice. Scalp hair loss is graded as mild, moderate, or severe; 4 secondary features each permit an increase of 1 severity level [32]. Photographs courtesy of Dr Kin Fon Leong (images for moderate and severe AA) and Freepik (image for mild AA). AA: alopecia areata.

Additionally, a recently developed Alopecia Areata Severity Scale offers a practical, clinic-friendly framework for grading AA. It uses the extent of scalp hair loss as the primary basis for a severity rating combined with 4 additional secondary clinical features, which, when present, permit an increase in the severity level (Figure 2) [32]. In local clinical practice, measures such as BSA involvement, the number of affected patches, serial photography, and overall clinical presentation are used to support assessment of disease severity and monitoring of treatment response. Standardized disease-assessment tools are recommended to guide care, monitor response, facilitate clinical decision-making, and support access or reimbursement discussions.

## Recognition of Clinical Burden and Psychosocial Impact of AA

AA presents a notable clinical burden in Malaysia, both in terms of treatment and its impact on patients’ quality of life [18]. Khoo et al [21] studied the psychosocial impact of alopecia in 240 patients with nonscarring alopecia attending a tertiary outpatient dermatology clinic from mid-2021 to mid-2022 in Penang, Malaysia. The median age was 38.5 (range 18‐70) years, the majority were of Malay ethnicity (42.5%), and 18.8% of patients were diagnosed with AA [21]. Most patients expressed significant concern about their hair loss, with younger individuals and unmarried respondents reporting higher levels of anxiety, depression, and impaired quality of life and female patients expressing greater concern about hair loss than male patients [21]. These findings emphasize that the psychosocial burden of alopecia is profound and requires targeted interventions in clinical practice [21].

Albela et al [14] recognized that AA can substantially affect health-related quality of life, particularly mental health. Patients with AA are also at increased risk of psychiatric comorbidities, including anxiety, depression, social phobia, and personality disorders, compared with the general population.

These findings highlight the complex emotional toll of AA and the importance of addressing mental health as a core component of patient care. Integrating psychological support into clinical management is essential to improve outcomes and provide more holistic care for patients.

Sex has been proposed as a factor in the psychosocial experience of AA, although the available evidence does not indicate a consistent difference in burden between male and female. In the local study by Khoo et al [21], female patients expressed greater concern about their hair loss than male patients, but there was no significant difference between male and female patients in Dermatology Life Quality Index or Hospital Anxiety and Depression Scale scores. A plausible explanation for the present observation is that men’s attitudes toward their hair and appearance are now comparable to those of women. Male patients should therefore be regarded as equally concerned and equally vulnerable, and their emotional burden should not be assumed to be lesser. In the cohort reported by Tang et al [15], 41% of patients did not return after the initial consultation, but this loss to follow-up was not stratified by gender, nor were the underlying reasons, such as inadequate treatment response, cost, or accessibility, systematically captured. The management of AA may also benefit significantly from a multidisciplinary, team-based approach that addresses both the physical and psychological effects of AA. Beyond dermatological treatment, individuals with AA often experience significant emotional and psychosocial challenges, including anxiety, depression, and reduced self-esteem [6,8]. Integrating mental health professionals, such as counselors or psychologists, into the care team may help provide holistic support [6]. Collaborative care models that bring together dermatologists, mental health specialists, and primary care providers could enhance patient outcomes. As such, there is a critical need to strengthen the integration of mental health services within existing AA management frameworks, promoting psychological intervention and ongoing support as standard components of care.

## Current Treatment Landscape and Implications for Targeted Therapies

Managing AA remains challenging due to unpredictable disease progression, variable treatment response, and high recurrence rates, especially with systemic steroids [33,34]. Additionally, long-term use of immunosuppressants such as methotrexate and azathioprine raises safety concerns [35,36].

The primary treatment modalities for AA in Malaysia range from corticosteroids (topical, oral or intralesionals), immunosuppressive therapy to phototherapy in selected hospitals, depending on age, subtype, and severity [20]. Yin and Chew [22] reported the use of topical steroids in more than half of 44 patients with AA in Malaysia (53.3%), followed by intralesional steroids (26.7%), topical minoxidil (17.8%), oral steroids (11.1%), oral finasteride (2.2%) and azathioprine (used concurrently with oral steroids) in 2.2% of patients. According to the retrospective 5-year audit at Hospital Kuala Lumpur between 2011 and 2015, AA accounted for 0.7% of cases treated with phototherapy [13]. Tang et al [15] found that topical corticosteroids were the most commonly used treatment, although other therapies, such as topical agents and phototherapy, were also used. However, the study also noted that 41% of patients did not return for follow-up care after the initial consultation, possibly suggesting challenges with treatment adherence. Ang et al [20] reported a case series of 11 patients with AA treated with topical immunotherapy using squaric acid dibutyl ester (SADBE). The median age of the patients was 13 (IQR 9-33) years; the majority were female (73%) and of Malay ethnicity (55%). The response rates to SADBE application were better in less severe forms of AA (80%) than in severe forms of AA (66%), and 28% of patients did not respond to SADBE [20]. A retrospective case series by Albela et al [14] reviewed 13 pediatric patients (6 boys and 7 girls, aged 4-16 years) with severe AA treated with oral methotrexate administered once weekly, with a mean dose of 0.4 mg/kg, at a center from January 2019 to December 2020. The authors observed that 5 of 12 assessable patients experienced more than 50% regrowth, while the remaining 7 had treatment failure.

Current local management appears to rely heavily on corticosteroids and conventional therapies, with treatment approaches often shaped by availability, cost, clinician experience, and disease severity. Globally, intralesional steroids remain the most widely used treatment for AA across all severity levels, followed by topical steroids, oral steroids, topical minoxidil, and Janus kinase (JAK) inhibitors [37,38], although intralesional steroid injections are often avoided in younger children due to discomfort and pain [39]. However, recent trends indicate a decline in steroid and minoxidil use as JAK inhibitors are gaining preference among dermatologists [40]. European expert consensus prioritize JAK inhibitors as a first-line systemic treatment for nonacute AA, before considering alternative systemic agents such as cyclosporine or methotrexate [41].

The introduction of JAK inhibitors has transformed the treatment landscape, offering a tolerable and effective option for patients with severe AA [42,43]. JAK inhibitors have shown promising results in improving treatment outcomes with early intervention in severe AA [43]. While JAK inhibitors have changed the global treatment landscape for moderate to severe AA, affordability, reimbursement, and equitable access remain major barriers in Malaysia. Treatment access discussions should be positioned around medical need, disease burden, validated severity assessment, and patient-centered outcomes. Attention should also be given to the ongoing challenges surrounding the reimbursement and accessibility of novel treatment options, particularly advanced therapies such as JAK family kinase inhibitors. These treatments, while showing promise in clinical outcomes, remain financially out of reach for many patients due to limited insurance coverage.

The absence of clearly defined national clinical practice guidelines highlights the need for more robust management strategies to support dermatologists, including pediatric dermatologists, and patients in shared treatment decision-making. National clinical guidelines could provide evidence-based recommendations for diagnosis, severity assessment, referral, treatment sequencing, monitoring, psychosocial care, and access discussions.

In the absence of country- or region-specific AA disease management guidelines, international guidelines and consensus statements can guide disease assessment, appropriate therapy selection, and treatment duration [41,44-46].

## Awareness of AA as a Chronic Immune-Mediated Disease

AA is not merely a cosmetic condition; it imposes a significant psychosocial burden, often resulting in social isolation, impaired productivity, and reduced quality of life [6,8,47,48]. Additionally, AA is linked to systemic conditions such as thyroid dysfunction and atopic dermatitis[4]. The public, medical professionals, and decision-makers must recognize that an active, immune-driven process leads to ongoing hair loss and distress in AA, and patients desperately seek treatments that halt disease progression and restore their original appearance [49,50]. Reframing AA as a chronic inflammatory disease rather than just a cosmetic concern could help drive policy changes and improve treatment accessibility [48].

Increased awareness of and advocacy about AA among the general population are needed through public education, which may aid in combating misconceptions about AA [18]. Hair fulfills both functional (eg, loss of eyelashes predisposes to eye irritation) and aesthetic roles and is crucial to self-image, self-expression, identity, and social perception, and its loss has been linked to increased anxiety, depression, and a diminished quality of life, particularly among younger individuals [21,51]. As a result, many patients turn to nonprescription and alternative treatments, a trend reflected by the fast-growing market for such products and supported by annual expenditures of US $30.2 billion on alternative medicine [21]. Khoo et al [21] noted that a significantly higher proportion of Malay patients opted for nonprescription and alternative therapies compared with other ethnic groups, suggesting hesitancy to try prescription medications. A common reason for avoiding consultation was the perception that hair loss is a natural part of aging, cited by 35.7% of respondents. Additionally, almost half of the patients (46.5%) reported dissatisfaction with their consultations, primarily due to the lack of specific treatment recommendations and unanswered questions [21]. Recognizing these attitudes and behaviors is essential for physicians and policymakers to improve consultation strategies and tailor interventions that address alopecia’s psychological and physical dimensions. Awareness of AA among patients, hair care professionals, and health care providers and prompt referral to a dermatologist are also important [52,53]. The rapid rise of hair salons and centers offering unapproved therapies risks delaying proper medical management and undermining effective care [54]. Public education campaigns can help reframe AA as an autoimmune disease with physical and psychological impacts rather than a purely cosmetic issue [55].

In the Malaysian setting, the reluctance to seek medical consultation reported by Khoo et al [21] appeared to reflect a knowledge gap, with hair loss commonly regarded as a natural part of aging and uncertainty as to whether it constitutes a medical condition.

The findings highlight the need for health care initiatives that improve public education and raise awareness and understanding of AA, as low awareness and stigmatization may contribute to psychological distress, delayed care-seeking, and uncertainty among patients about whether AA is a medical condition requiring clinical management. Public and clinician awareness should emphasize that medical treatment options are available and that timely referral may support appropriate assessment and care.

## Conclusions

**Figure 3.**
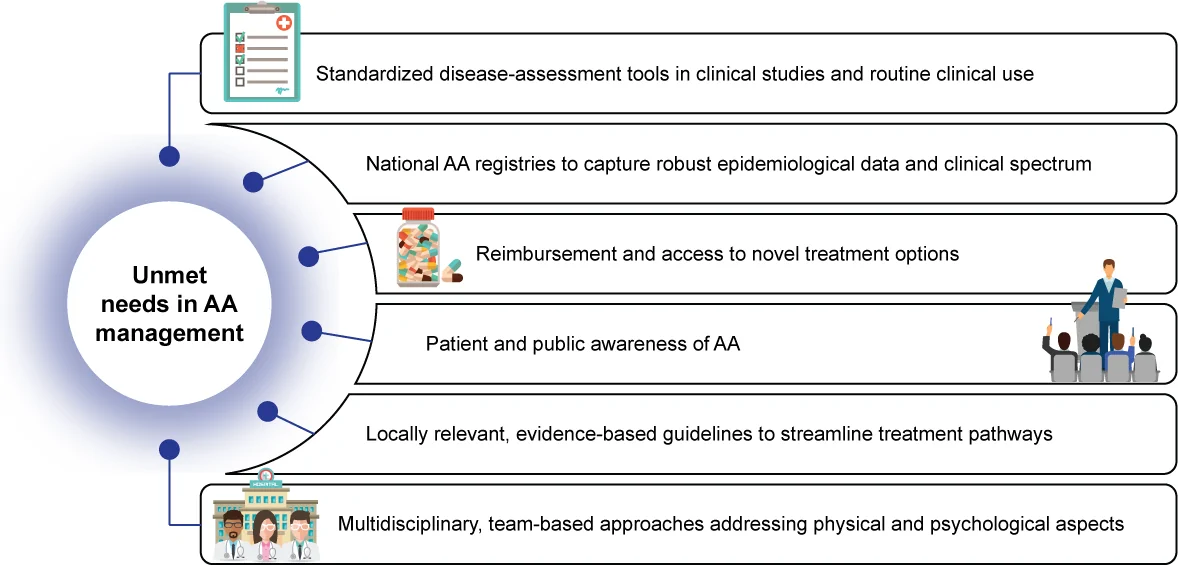
Unmet needs in the management of alopecia areata (AA) in Malaysia. Priority actions to address gaps in AA epidemiological data, severity assessment, treatment access, public awareness, and multidisciplinary care in Malaysia.

AA is a chronic, immune-mediated disease with substantial clinical and psychosocial impact, yet Malaysia-specific evidence remains limited and is largely derived from retrospective, single-center studies. Available local data suggest variable disease presentation, the need for standardized severity-assessment tools, reliance on conventional therapies, substantial loss to follow-up, and a meaningful psychosocial burden among affected patients. These findings highlight the need to strengthen local epidemiological and real-world evidence, adopt standardized disease-assessment tools in clinical studies and routine practice, and integrate psychosocial support into AA care pathways (Figure 3). Improving public and clinician awareness is also important to reframe AA as a medical condition rather than a cosmetic concern and to encourage timely referral and appropriate management. Looking ahead, locally relevant clinical guidance to support evidence-based management, multidisciplinary care models for patient-centered AA care, and equitable access to effective treatment options, including advanced therapies where appropriate, will be essential to improve outcomes for both adult and pediatric patients with AA in Malaysia.

## Supplementary material

10.2196/89583 Multimedia Appendix 1 Summary of the identified alopecia areata studies and literature from Malaysia.

## Abbreviations

AA: alopecia areata
AA-IGA: Alopecia Areata Investigator Global Assessment
AAPPO: Alopecia Areata Patient Priority Outcome
BSA: body surface area
EBA: Eyebrow Assessment Scale
ELA: Eyelash Assessment Scale
JAK: Janus kinase
PGI-C: Patient’s Global Impression of Change
SADBE: squaric acid dibutyl ester
SALT: Severity of Alopecia Tool
