# Supplementary material for: Current Management and Unmet Needs in Alopecia Areata: Viewpoint From a Malaysian Perspective
Source: JMIR Dermatol. 2026 Sep 21;9:e89583. doi: 10.2196/89583 (PMC13592563; doi:10.2196/89583)
Supplement: Supplementary file 1 [file derma-v9-e89583-s001.docx]

| **Table S1.** Summary of the identified alopecia areata studies and literature from Malaysia | | | | |
| --- | --- | --- | --- | --- |
| **Study** | **Objective (and study design)** | **Location, population and time period** | **Disease severity assessment of AA** | **Key findings and unmet needs for AA management** |
| **“Clinical Pattern, Management and Outcome of Alopecia Areata of a Multi-Ethnic Population in Kuala Lumpur Hospital, Malaysia”**  **Tang et al. [1]** | To study the demographic, clinical patterns, management strategies and outcomes (retrospective study). | Medical records of 200 multi-ethnic patients from Kuala Lumpur Hospital, Malaysia from January 1987 until June 2009.  Alopecia areata accounted for 197 (98.5%) the cases. | Not stated | All the patients received topical treatment for AA. Complete response was seen in less than 20% of patients.  The low complete response rate to topical treatments, seen in less than 20% of patients, highlights the need for more effective therapeutic options. |
| **“A 10-Year Retrospective Review of the Spectrum of Non-Scarring Alopecia in a Tertiary Hospital, Malaysia”**  **Lee et al. [2,3]** | To review the clinical characteristics and treatment given to non-scarring alopecia patients (retrospective study). | Medical records of 156 patients at University Malaya Medical Centre from January 2000 until December 2009.  Alopecia areata accounted for 45 (28.7%) of the cases. | Not stated | AA was the most common cause of non-scarring alopecia.  This highlights the need for targeted management strategies and increased awareness among healthcare providers. |
| **“The Utilization of Phototherapy in the Department of Dermatology, Hospital Kuala Lumpur: A 5-year audit”**  **Vaani et al. [4]** | To describe the utilization of phototherapy in the Department of Dermatology Hospital Kuala Lumpur (retrospective study). | Medical records of 892 patients attending the Department of Dermatology Hospital Kuala Lumpur between 2011 and 2015  Alopecia areata accounted for 6 (0.7%) of the cases | Not stated | Narrow-band UVB was the most frequently prescribed phototherapy at the center.  The reliance on narrow-band UVB as the primary phototherapy highlights a need for broader access to other treatment modalities and individualized care approaches. |
| **“Non-Ionizing Radiation Exposure Causing Ill-Health and Alopecia Areata”**  **Isa et al. [5]** | To present cases of occupational exposure to radio-frequency and microwave radiation (case series). | Case reports of two 38-year-old men with AA from the Hospital Universiti Sains Malaysia in December 1990. | Not stated | The exposure threshold, safety standards, and biological effects of the non-ionizing radiation need to be further studied for their role in causing AA |
| **“Alopecia Areata”**  **Kaur et al. [6]** | To elaborate the process of diagnosis and treatment of individuals with AA (case report). | Case report of a 55-year-old Malaysian Chinese man presenting with AA in October 2021. | - The pull test | Intralesional triamcinolone acetonide administered over 2  months, along with prednisone, minoxidil, and vitamin C. Full treatment took <6 months as AA was addressed in the early stages of the disease, highlighting the importance of early diagnosis and treatment. |
| **“Efficacy and Tolerability of Methotrexate in the Treatment of Severe Paediatric Alopecia Areata”**  **Albela et al. [7]** | To study the efficacy and tolerability of MTX in severe pediatric AA (retrospective case series). | Thirteen patients (6 boys and seven girls) aged between 4 and 16 years with severe refractory AA from the Department of Paediatrics, Women & Children Hospital, Kuala Lumpur, between January 2019 and December 2020. | - The extent of hair loss was categorized as follows: S1, <24%; S2, 25–49%; S3, 50–74%; S4, 75– 99%; and S5, 100%. - Regrowth was categorized as follows: A0, no change or further loss; A1, 1–24%; A2, 25–49%; A3, 50–74%; A4, 75–99%; and A5, 100% | 7 of 12 assessable patients were treatment failures with MTX. MTX showed variable efficacy for treating paediatric AA with overall good tolerability. This highlights the need for the access to wider treatment options for managing pediatric AA. |
| **“A Pediatric Case of Severe Conjunctivitis with Underlying Alopecia Areata”**  **Azlan et al. [8]** | To present the case of a 7-year-old girl who was under investigation for AA (case report). | Case report of a 7-year-old Malaysian girl from the University Malaya Medical Centre | Not stated | Highlights the comorbidities in AA, and in this case, bilateral eye allergic  Conjunctivitis, bilateral cornea scarring and failure to thrive, which remained undiagnosed and untreated despite previous visits to medical centres. |
| **“Prevalence of Skin Diseases in Dermatology Outpatient**  **Clinic, Hospital Kuala Lumpur.”**  **Heah et al. [9]** | To determine the prevalence and changing trend of skin diseases according to age, gender and ethnicity in Hospital Kuala Lumpur (retrospective cross sectional study). | Department of Dermatology, Hospital Kuala Lumpur from 1^st^ January 2008 to 31^st^ December 2014  Overall, 58,252 patients consisting of Malays (61.0%), Indians (20.1%) and Chinese (18.9%) | Not stated | Prevalence of AA was 1.32% in 58,252 patients seen over 7 years. Malays represented 69.2% of AA cases, followed by Indians 28% and Chinese 10%. |
| **“Efficacy of Topical Immunotherapy with Squaric Acid Dibutylester in the Treatment of Alopecia Areata – A Case Series of 11 Patients.”**  **Ang et al. [10]** | To determine squaric acid dibutyl ester (SADBE) treatment outcome and side effects among AA patients at the Dermatology Department, Hospital Raja Permaisuri Bainun, Ipoh, Malaysia (retrospective cross-sectional single centre study). | Data was collected from 11 patients with AA from the Dermatology Department, Hospital Raja Permaisuri Bainun, Ipoh, Malaysia between December 2015 and July 2017. | Clinical response was determined by McDonald Hull and Norris’s grading system as follows: Grade 1 - Regrowth of vellus hair   - Grade 2 - Regrowth of sparse pigmented terminal hair - Grade 3 - Regrowth of terminal hair with patches of alopecia - Grade 4 - Regrowth of terminal hair on the scalp | 8 of 11 AA patients achieved a good response. Three patients also had side effects including persistent irritation, vesicle and blister formation.  Patients with varying severity of AA require reliable and safe treatment options, emphasizing the need for therapies that are both effective and well-tolerated with minimal adverse effects. |
| **“Attitudes, Health Seeking**  **Behaviours, Expectations and Psychosocial Impact of Patients with Non-Scarring Alopecia: Results of Tertiary Outpatient Skin Specialist Clinics in Penang”**  **Khoo et al. [11]** | To characterize the concerns, health care seeking behaviours, expectations  and psychosocial impact of patients with alopecia, plus the contributing factors (questionnaire-based cross-sectional study). | 240 patients with AA from the Dermatology Clinic in Hospital Pulau Pinang  from July 2021 to June 2022. | N/A | There is an unmet need for psychological support and interventions for patients with alopecia, particularly younger individuals, unmarried patients, and those who are employed, as hair loss is associated with significant anxiety, depression, and reduced quality of life. |
| AA, Alopecia Areata; MTX, methotrexate; SADBE, squaric acid dibutylester; UVB, ultraviolet B radiation | | | | |

**References**

1. Tang MM, Gill P, Thevarajah S. Clinical pattern, management and outcome of alopecia areata of a multi-ethnic population in Kuala Lumpur Hospital, Malaysia. *Exp Dermatol*. 2010;19:600. URL: <https://www.ovid.com/journals/exder/abstract/00023629-201006000-00157~clinical-pattern-management-and-outcome-of-alopecia-areata> [Accessed 2026-09-03]
2. Lee YY, Lee CK. A 10-year retrospective review of non-scarring alopecia in a tertiary hospital, Malaysia. *Exp Dermatol*. 2010;19(6 Suppl):600. [doi: [10.1111/j.1600-0625.2010.01097.x](https://onlinelibrary.wiley.com/doi/10.1111/j.1600-0625.2010.01097.x)]
3. Yin YL, Chew KL. A 10-year retrospective review of non-scarring alopecia in a tertiary hospital in Malaysia. *Malays J Dermatol*. 2012;29(2):12-5. URL: <https://www.dermatology.org.my/pdf/journal%202012%20B1.pdf> [Accessed 2026-09-14]
4. Vaani VV, Tang MM, Tan LL, Asmah J. The utilization of phototherapy in the department of dermatology, Hospital Kuala Lumpur: a 5-year audit. *Med J Malaysia*. Jun 2018;73(3):125-130. [Medline: [29962494](https://pubmed.ncbi.nlm.nih.gov/29962494/)]
5. Isa AR, Noor M. Non-ionizing radiation exposure causing ill-health and alopecia areata. *Med J Malaysia*. Sep 1991;46(3):235-238. [Medline: [1839918](https://pubmed.ncbi.nlm.nih.gov/1839918/)]
6. Kaur I. Alopecia areata. *Clin Dermatol J*. 2022;7(2). [doi:[10.23880/CDOAJ-16000274](https://medwinpublishers.com/CDOAJ/alopecia-areata.pdf)]
7. Albela H, Begum S, Wee AL, Ponnuthurai N, Leong KF. Efficacy and tolerability of methotrexate in the treatment of severe paediatric alopecia areata. *Skin Appendage Disord*. May 2022;8(3):206-210. [doi: [10.1159/000521238](https://pubmed.ncbi.nlm.nih.gov/35707294/)] [Medline: [35707294](https://pubmed.ncbi.nlm.nih.gov/35707294/)]
8. Azlan A, Khaliddin NB. A paediatric case of severe allergic conjunctivitis with underlying alopecia areata. *Malays J Med Res*. 2023;07(1):19-21. [doi: [10.31674/mjmr.2023.v07i01.004](https://ejournal.lucp.net/index.php/mjmr/article/view/1812)]
9. Heah SK, Mohd Noor N, Johar A. Prevalence of skin diseases in dermatology outpatient clinic, Hospital Kuala Lumpur*. Malays J Dermatol*. 2017;38:19-24. URL: <https://www.dermatology.org.my/pdf/MJD%20June%202017%20Issue%2038.pdf> [Accessed 2026-09-03]
10. Ang TS, Tang JJ. Efficacy of topical immunotherapy with squaric acid dibutylester in the treatment of alopecia areata – a case series of 11 patients. *Malays J Dermatol*. 2018;40:65-68. URL: <https://www.dermatology.org.my/pdf/Malaysian_Journal_of_Dermatology_2018_05Jul18.pdf> [Accessed 2026-09-08]
11. Khoo VL, Loo CH, Khor YH, Mohd Ali N, Tan WC. Attitudes, health seeking behaviors, expectations and psychosocial impact of patients with non-scarring alopecia: results of tertiary outpatient skin specialist clinics in Penang. *Malays J Dermatol*. 2023;50:29-39. URL:<https://www.dermatology.org.my/pdf/MJD_Jun2023.pdf> [Accessed 2026-09-08]
